# HCV elimination: is the bulk of the iceberg being missed?

**DOI:** 10.1093/gastro/goae093

**Published:** 2024-10-15

**Authors:** Eric Kalo, Asma Baig, Alison Derrett, Scott Read, Golo Ahlenstiel

**Affiliations:** Blacktown Clinical School, School of Medicine, Western Sydney University, Blacktown, NSW, Australia; Blacktown Mount Druitt Hospital, Western Sydney Local Health District, Blacktown, NSW, Australia; Blacktown Mount Druitt Hospital, Western Sydney Local Health District, Blacktown, NSW, Australia; Western Sydney Local Health District, Westmead, NSW, Australia; Blacktown Clinical School, School of Medicine, Western Sydney University, Blacktown, NSW, Australia; Blacktown Mount Druitt Hospital, Western Sydney Local Health District, Blacktown, NSW, Australia; Storr Liver Centre, The Westmead Institute for Medical Research, University of Sydney, Westmead, NSW, Australia; Blacktown Clinical School, School of Medicine, Western Sydney University, Blacktown, NSW, Australia; Blacktown Mount Druitt Hospital, Western Sydney Local Health District, Blacktown, NSW, Australia; Western Sydney Local Health District, Westmead, NSW, Australia; Storr Liver Centre, The Westmead Institute for Medical Research, University of Sydney, Westmead, NSW, Australia

## Introduction

In 2016, the Australian government spent more than 1 billion dollars to make hepatitis C virus (HCV) direct-acting antivirals (DAAs) available to all as part of a promising plan to eradicate HCV within a generation [1]. Moreover, Australia's unique approach of offering well-tolerated pan-genotypic DAAs (sofosbuvir/velpatasvir, glecaprevir/pibrentasvir) through general practitioners has significantly increased treatment accessibility. As a result, ∼60% of Australians who are living with hepatitis C have received treatment. While significant progress has been made, reaching the 2030 elimination goal poses a challenge that is both feasible and attainable [2–4].

Current testing strategies are not comprehensive enough to fully identify all HCV-infected people. Current screening in Australia targets high-risk populations, including people who inject drugs, those of indigenous descent, those who take part in high-risk sexual behavior, or those with current or a history of incarceration. Such targeted screening strategies continue to enforce stigma and allow discrimination to linger in community and healthcare settings. Indeed, >70% of people living with HCV in Australia have reported experiencing stigma and discrimination, including withholding treatment, diagnostic overshadowing, unwelcoming, and/or excessive infection control behaviors [5]. In fact, current screening is not comprehensive enough to fully identify all HCV-infected people.

The aim of this study is to assess HCV prevalence within non-risk-factor populations in our community who are attending Blacktown and Mt Druitt hospitals’ outpatient services in Western Sydney, New South Wales, Australia.

## Methods

Electronic medical records of four cohorts of adult patients who attended testing for a non-risk-factor-related reason in antenatal, oncology, renal dialysis, and metabolic outpatient clinics between 2019 and 2021 (*n *=* *11,006) were reviewed for point-of-care HCV antibody testing with reflex testing for HCV RNA. Other parameters, such as blood-borne virus testing, Fibrosis-4 (FIB-4) index for liver fibrosis, aspartate aminotransferase to platelet ratio index (APRI), demographics, and risk-factor data, were also collected.

Ethics approval was obtained from the Human Research Ethics Committee at Western Sydney Local Health District (Ethics Approval 2021/ETH00149). The study conforms to the ethical guidelines of the Declaration of Helsinki and Istanbul.

## Results

The prevalence of HCV antibody seropositive individuals was 0.64% in antenatal clinics, 3.87% in oncology clinics, 2.34% in dialysis clinics, and 1.16% in metabolic clinics (Supplementary Table 1). An average prevalence was 0.76%, compared with the documented New South Wales prevalence of 0.04% in 2019 and 0.037% in 2020 [6]. Following the initial diagnosis, 84% of all positive cases had further HCV RNA testing and 6% had subsequent management. Among individuals who tested positive for HCV antibodies, 64% had an APRI score of <0.5 and 77% had a FIB-4 score of <1.45. Consistently with current testing strategies, risk factors for HCV infection comprised 46% for people who injected drugs and 18% for individuals of indigenous descent.

## Discussion

**Figure 1.**
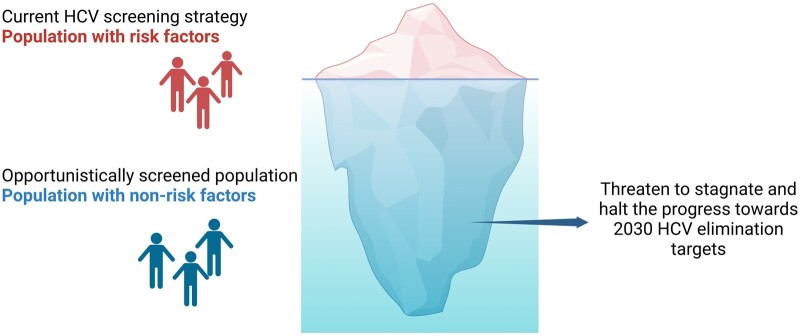
HCV elimination: is the bulk of the iceberg being missed? Current risk-factor-based screening strategy fails to capture a significant number of HCV-infected people, potentially hindering or even halting progress toward the 2030 HCV elimination targets.

Although Australia has made significant progress towards hepatitis C elimination, our study suggests that a considerable number of HCV-infected patients are overlooked when using the risk-factor screening approach. Notably, all our cohorts had significantly higher prevalence of HCV cases as compared with the estimated New South Wales prevalence. Nearly 54% of patients had a known risk factor identified and 75% did not exhibit any clinically significant increase in liver enzymes or markers of fibrosis. Thus, such patients are typically not flagged for HCV screening in general practice outside of risk-factor-based approaches. This would suggest that a screening approach that is not based on risk factors is superior at identifying “hidden” cases of undiagnosed HCV. In addition to the incarcerated population, this “hidden” group of HCV carriers may threaten to stagnate and even halt the progression towards the 2030 elimination targets in Australia (Figure 1).

This study further suggests that the expansion of screening strategies to include non-risk populations may be necessary. A rethinking of our current risk-factor-based models of care may also help in normalizing testing practice and curbing stigma and persistent discrimination in healthcare settings.

Although the development of DAAs has greatly promoted the progress of HCV elimination, there cannot be any doubt that effective screening programs and high treatment uptake will be crucial to finally achieving global HCV elimination. Moreover, different point-of-care strategies need to be developed to guide early HCV detection and enhance decentralization of care [7]. Programs that use rapid diagnostic tests (such as dried blood spot testing, point-of-care antibody and RNA testing, reflex RNA testing, and opt-out screening) and immediate initiation of antiviral treatment will not only improve the early diagnosis of HCV infection, but also facilitate linkage to care and treatment among different populations and settings [8–10].

## Supplementary Material

goae093_Supplementary_Data
