# Supplementary material for: HCV elimination: is the bulk of the iceberg being missed?
Source: Gastroenterol Rep (Oxf). 2024 Oct 15;12:goae093. doi: 10.1093/gastro/goae093 (PMC11479704; doi:10.1093/gastro/goae093)
Supplement: goae093_Supplementary_Data [file goae093_supplementary_data.docx]

**Table 1.** Prevalence of HCV infection within populations accessing outpatient services (antenatal, oncology, dialysis and metabolic clinics) at Blacktown and Mt Druitt Hospitals, Western Sydney, NSW, Australia

| **Total tested** | **Patients (*N* = 11,006)** |
| --- | --- |
| Total HCV Ab (+) | 84 (0.76%) |
| **Gender** |  |
| Female | 68 |
| Male | 16 |
| **Age, years, IQR** | 35 (22, 83) |
| **FIB-4 score, *n*** |  |
| Mild (<1.45) | 66 |
| Moderate (1.45–3.23) | 10 |
| Severe (>3.25) | 2 |
| **Antenatal cohort (per 100,000)** |  |
| HCV Ab (+) | 0.64% |
| **Oncology cohort (per 100,000)** |  |
| HCV Ab (+) | 3.87% |
| **Dialysis cohort (per 100,000)** |  |
| HCV Ab (+) | 2.34% |
| **Metabolic cohort (per 100,000)** |  |
| HCV Ab (+) | 1.16% |

Ab, antibody; FIB-4, index for liver fibrosis; HCV, hepatitis C virus.
